# Effects of *in ovo* Inoculation of Multi-Strain Lactobacilli on Cytokine Gene Expression and Antibody-Mediated Immune Responses in Chickens

**DOI:** 10.3389/fvets.2020.00105

**Published:** 2020-02-28

**Authors:** Mohammadali Alizadeh, Bahram Shojadoost, Jake Astill, Khaled Taha-Abdelaziz, Seyed Hossein Karimi, Jegarubee Bavananthasivam, Raveendra R. Kulkarni, Shayan Sharif

**Affiliations:** ^1^Department of Pathobiology, Ontario Veterinary College, University of Guelph, Guelph, ON, Canada; ^2^Department of Pathology, Faculty of Veterinary Medicine, Beni-Suef University, Beni Suef, Egypt; ^3^Department of Pathology and Molecular Medicine, McMaster Immunology Research Centre, M. G. DeGroote Institute for Infectious Disease Research, McMaster University, Hamilton, ON, Canada; ^4^Department of Population Health and Pathobiology, College of Veterinary Medicine, North Carolina State University, Raleigh, NC, United States

**Keywords:** lactobacilli, *in ovo*, chickens, cytokines, antibody

## Abstract

This study was conducted to investigate the effects of various doses of a multi-strain lactobacilli mixture (*Lactobacillus salivarius, Lactobacillus reuteri, Lactobacillus crispatus*, and *Lactobacillus johnsonii*) on the innate and adaptive immune responses in broiler chickens. At embryonic day eighteen, 200 eggs were injected with PBS, or three different doses of a multi-strain lactobacilli mixture (1 × 10^5^, 1 × 10^6^, and 1 × 10^7^ CFU/egg, P1, P2, and P3 respectively) along with a group of negative control. On days 5 and 10 post-hatch, cecal tonsil, bursa of fabricius, and spleen were collected for gene expression and cellular analysis. On days 14 and 21 post-hatch, birds were immunized intramuscularly with both sheep red blood cells (SRBC) and keyhole limpet hemocyanin (KLH). Serum samples were collected on days 0, 7, 14, and 21 after primary immunization. The results demonstrated that lactobacilli inoculation increased the splenic expression of cytokines, including interferon (IFN)***-***α, IFN*-*β, IFN*-*γ, interleukin (IL)-8, and IL-12 on day 5 post-hatch compared to the control group (PBS). However, in cecal tonsils, lactobacilli treatment downregulated the expression of IL-6 on day 5 post-hatch and IL-2 and IL-8 on day 10 post-hatch. No significant differences were observed in the expression of cytokine genes in the bursa except for IL-13 which was upregulated in lactobacilli-treated groups P2 and P3 on days 5 and 10 post-hatch. Flow cytometry analysis showed that the percentage of KUL01, CD4^+^ and CD8^+^ splenocytes was not affected by treatments. In addition, no significant differences were observed for antibody titers against SRBC. However, lactobacilli treatment (P1, P2, and P3) was found to increase IgM titers on day 21 post-primary immunization compared to controls. Furthermore, *in ovo* injection of the highest dose of probiotics (1 × 10^7^, P3) increased serum IgG titers against KLH on day 7 post-primary immunization. In conclusion, this study demonstrated that that *in ovo* administration of lactobacilli can improve antibody-mediated immune responses and differentially modulate cytokine expression in mucosal and systemic lymphoid tissues of chickens.

## Introduction

In the poultry industry, it is common for newly hatched chickens to experience delayed access to feed and water due to the time spent in the hatchery and during transportation to the production farm (1). This delay in feed and water intake may negatively influence post-hatch immune system function and bird performance (2). In addition, in broiler chickens, parents do not contribute to egg incubation, and development of the embryo occurs independently of its mother reducing parental influence on gut microbial development (3). Gut microbiota provides essential health benefits to the host by enhancing immune system development and maintaining and regulating intestinal immune homeostasis (4, 5). Recent studies have suggested that dysbiosis in gut microbiota is linked to the pathogenesis of a variety of intestinal disorders (6, 7). In chickens, the establishment of the gut microbiota occurs within 3 days post-hatch and the microbial composition remains relatively unchanged until 30 days of age (8). This indicates that early establishment of beneficial bacteria is very important and can further impact gut microbiota colonization and the development of barrier functions of the gastrointestinal tract (9–11). Therefore, pre-hatch colonization of chickens' gastrointestinal tracts with beneficial bacteria through *in ovo* technology may prevent pathogen colonization via competitive exclusion in addition to accelerating intestinal and immune system development (10). Different studies have reported the beneficial effects of probiotic bacteria on broiler growth performance, gut microbiota composition and immune system development (12–15). Among these probiotics, *Lactobacillus* bacteria have received considerable attention because of their immunomodulatory activities and intestinal health benefits (16–18). Lactobacilli are considered autochthonous residents in the chicken gastrointestinal tract and may contribute to the host gut health and immune system function through different mechanisms such as enhancement of the epithelial barrier, competitive exclusion of pathogenic microorganisms, production of antimicrobial substances, and interaction with immune system cells via stimulation of pattern recognition receptors (19, 20). Considering the vulnerability of newly hatched chicks toward various pathogens, pre-hatch administration of *Lactobacillus* bacteria via *in ovo* technology can be used as a strategy to strengthen immune responsiveness of chickens and reduce their susceptibility toward pathogens. Many studies suggest that different strains of lactobacilli can modulate multiple aspects of immune response including cytokine and chemokine expression, T lymphocyte populations and systemic antibody-mediated responses (21–23). In the present study, we hypothesized that one-time *in ovo* administration of a mixture of four *Lactobacillus* spp. (*L*. *salivarius, L*. *reuteri, L*. *crispatus*, and *L. johnsonii)* can modulate innate responses and thus, can accelerate the maturation of the immune system leading to enhanced antibody-mediated responses against thymus-dependent antigens. Therefore, this study was aimed at investigating the potential immunomodulatory effects of *in ovo* administration of lactobacilli on innate and antibody-mediated immune response in chickens.

## Materials and Methods

### Chickens and Housing

Embryonated chicken eggs were obtained from the Arkell Poultry Research Hatchery (University of Guelph, ON, Canada). Newly hatched commercial broiler chicks housed in a separated floor pens per each treatment group, on clean wood shavings with free access to water and feed at Arkell Poultry Research.

### Experimental Design

In this experiment, the selected *Lactobacillus* spp. including *L. salivarius, L. reuteri, L. crispatus*, and *L. johnsonii* were isolated from the intestinal contents of healthy broiler chickens as previously described (16). Two hundred embryonated broiler chicken eggs were incubated at 37°C at Arkell Research Station (Guelph, ON). On day 18 of incubation, 40 embryonated eggs were injected with one of three different doses of a selected mixture of *Lactobacillus* bacteria, including 1 × 10^5^ CFU (P1), 1 × 10^6^ CFU (P2), and 1 × 10^7^ CFU (P3) of bacteria or phosphate buffered saline (PBS), all injections were 100 μL total volume. Each *lactobacillus* was grown separately and prepared at the certain dose from 1 × 10^5^ to 1 × 10^7^ cfu/ml in PBS and the strains were associated in equal amount within the multi-strain cocktail designated for this study. The remaining eggs (24) served as a non-injected untreated negative control, creating 5 groups. The lactobacilli cocktail was delivered precisely to amniotic fluid, where the negative pressure in abdominal cavity facilitates the passage of the intestinal content via peristaltic movement. Lactobacilli used in the present study have been recovered from the intestines of newly hatched chickens (unpublished data). This was assessed using a culture-based method and would be relevant to use in the future to use tagged bacteria for tracking them in the intestine.

### Immunization, Serum Collection, and Tissue Sampling

To evaluate antibody-mediated immune responses, on days 14 and 21 post-hatch, birds were immunized intramuscularly with 0.25 mL of 2% SRBC (PML Microbiologicals, Mississauga, ON, Canada) in PBS and subsequently with 0.25 ml of PBS containing 100 μg keyhole limpet hemocyanin (KLH) (Sigma, Oakville, ON, Canada). The untreated, unimmunized group was injected with PBS. Blood samples (1–2 ml) were collected from the wing vein of 12 birds per treatment on days 0, 7, 14, and 21 post primary immunization. Blood samples were kept at room temperature for 2 h and then centrifuged at 580 × *g* for 10 min to isolate serum. Serum samples were stored at –20°C for antibody analysis. On days 5 and 10 post-hatch 6 birds per treatment were euthanized and bursa of Fabricius, cecal tonsils, and spleen tissues were collected, kept in RNA later and stored at −80°C for gene expression analysis. Spleen tissue was also kept on ice in 1 X Hanks' balanced salt solution (HBSS) (Gibco, Grand Island, NY) for analysis of splenocytes with flow cytometry.

### Isolation of Spleen Mononuclear Cells and Flow Cytometry Analysis

Single-cell suspensions of mononuclear cells were prepared according to the procedure of Taha-Abdelaziz et al. (25). Briefly, spleen samples from 6 chickens per treatments were rinsed three times in HBSS and filtered through a 40-μm nylon cell strainer using the flat end of a 1 ml syringe plunger. Cells were resuspended in 5 ml RPMI (Invitrogen, Burlington, Ontario, Canada) containing 10% fetal bovine serum, 2.5% HEPES (Sigma Aldrich, St Louis, MO), 1% Penicillin-Streptomycin (Gibco, Grand Island, NY), 0.5% Gentamicin (Gibco, Grand Island, NY), and 0.05% 2-Mercaptoethanol (Sigma Aldrich, St Louis, MO) and they were overlaid on 4 ml Histopaque-1077 (Sigma, Oakville, ON) for density gradient separation, and mononuclear cells at the interface were harvested and washed twice in RPMI (Gibco, Grand Island, NY) media. Cells were counted using automated cell counter MOXI Z (Orflo, Ketchum, ID, USA) and 100 μL of each cell suspension was seeded in round bottom 96 well plates at density of 1 × 10^6^ /ml in RPMI medium. Subsequently, cells were washed twice in FACS buffer (PBS containing 1% BSA) and stained for 30 min at 4°C in the dark with fluorescent monoclonal antibodies including mouse anti-chicken CD3-PB [CT-3], mouse anti-chicken CD4-PE [CT-4], mouse anti-chicken CD8-APC [CT-8], and mouse anti-chicken monocyte/macrophage-FITC [KUL01] (Southern Biotechnology Associates, Inc., Burlington, ON). The cells were washed twice in FACS buffer, fixed in 2% paraformaldehyde (PFA) and transferred to 5 ml polystyrene round-bottom tubes for analysis. Flow cytometry was performed using a FACS Canto II flow-cytometer (BD Bioscience, San Jose, CA, USA) and data were analyzed using FlowJo Software (v.10).

### Serological Analysis

Detection of the total antibody responses to SRBC in sera was performed by a direct hemagglutination assay according to the procedure of Haghighi et al. (26). Serum samples were heat-treated at 56°C for 30 min. Then, 50 μL of PBS containing 0.05% of bovine serum albumin (BSA) was added into each well of a round-bottomed 96-well microplate, and 2-fold serial dilutions of serum samples were generated in duplicate. Subsequently, 50 μL of 1% SRBC in PBS was added to each well and the plates were shaken for 1 min followed by incubation for 24 h at 37°C. Positive result were recorded when at least 50% of SRBC agglutination was observed.

Detection of KLH-specific IgG and IgM titers in sera was performed by indirect enzyme-linked immunosorbent assay (ELISA). Briefly, each well of a flat-bottomed 96-well Maxisorp high binding microplate was coated overnight at 4°C with 100 μL of 1 μg/ml KLH in coating buffer (0.1 M NaHCO_3_, pH 9.6) containing BSA (30 μg /ml). Wells were then washed 4 times with 200 μL of PBS with 0.05% Tween 20 (P137 Sigma Aldrich Inc., St. Louis, MO) (PBST) and were completely decanted between each washing step. Subsequently, 100 μL of blocking buffer (PBST containing 0.25% of gelatine) was added to each well and the plate was incubated for 2 h at room temperature. Washing was repeated and was followed by addition of 100 μL of chicken serum (diluted 1:200 v/v in blocking buffer) to each well. Plates were incubated 2 h at room temperature and then were washed 4 times with the washing solution. One hundred μL of detection antibody (goat anti-chicken IgG-Fc and IgM-Fc, Bethyl laboratories) conjugated with horseradish peroxidase (diluted in 1/5,000 of blocking buffer) was added to each well and incubated for 1 h at room temperature. Washing was repeated and was followed by addition of 100 μL ABTS [2,2_-azinobis (3 ethylbenzthiazolinesulfonic acid)] peroxidase substrate system (Mandel Scientific, Guelph, ON, Canada) to each well. Plates were incubated for 30 min at room temperature in the dark and absorbance was measured at 405 nm using the micro plate reader (Epoch, BioTek Instruments Inc., Winooski, VT). Positive and negative-control serum (fetal bovine serum) were included in each plate to justify the plate-to-plate variations. Sample/positive (Sp) ratios were calculated according to the following formula: (mean of test sample—mean of negative control)/(mean of positive control—mean of negative control).

### RNA Extraction and Reverse Transcription

Total RNA was extracted from spleen, bursa of Fabricius and cecal tonsil tissues using Trizol as described by the manufacturer (Invitrogen Canada Inc., Burlington, ON, Canada). Total RNA was treated with DNase (DNA-free kit, Ambion, Austin, TX) and the quantity and purity of the RNA samples was measured by using a Nanodrop spectrophotometer (Thermo Scientific, Wilmington, DE). Reverse-transcription to cDNA was performed by using Superscript® II First Strand Synthesis kit (Invitrogen) according to the manufacturer's protocol.

### Quantitative Real-Time PCR

Quantitative real-time (qRT) PCR was performed using the LightCycler® 480 II system (Roche Diagnostics GmbH, Mannheim, DE). Each qRTPCR reaction consisted of 10 μl of 2X SYBR Green Master mix (Roche Diagnostics), 1 μl of forward- and 1 μl of reverse-primer (5 μM), 3 μl PCR-grade water and 5 μl of target cDNA (1:10, diluted in nuclease free-water). The PCR cycling protocol included an initial denaturation step at 95°C, followed by amplification for 40–50 cycles consisting of 95°C for 10 s, an annealing step at a temperature described in Table 1 for each of the primer pairs, and extension at 72°C for 10 s. The primers used were synthesized by Sigma-Aldrich (Oakville, ON), and their specific sequences and accession numbers are presented in Table 1.

**Table 1:** Primer sequences used for real-time quantitative PCR^a^.

**Gene^b^**	**Primer sequence^c^ (5^′^-3^′^)**	**Annealing temperature**	**GeneBank accession number**
IFN-α	F: ATCCTGCTGCTCACGCTCCTTCT	64	AB021154
	R: GGTGTTGCTGGTGTCCAGGATG		
IFN*-β*	F: GCCTCCAGCTCCTTCAGAATACG	64	AY974089
	R: CTGGATCTGGTTGAGGAGGCTGT		
IFN-γ	F: ACACTGACAAGTCAAAGCCGCACA	60	X99774
	R: AGTCGTTCATCGGGAGCTTGGC		
IL-2	F: TGCAGTGTTACCTGGGAGAAGTGGT	60	NM_204153.2
	R: ACTTCCGGTGTGATTTAGACCCGT		
IL-6	F: CGTGTGCGAGAACAGCATGGAGA	60	NM_204628.1
	R: TCAGGCATTTCTCCTCGTCGAAGC		
IL-8	F: CCAAGCACACCTCTCTTCCA	64	AJ009800
	R: GCAAGGTAGGACGCTGGTAA		
IL-12p40	F: CCAAGACCTGGAGCACACCGAAG	60	AY262752.1
	R: CGATCCCTGGCCTGCACAGAGA		
IL-13	F: ACTTGTCCAAGCTGAAGCTGTC	60	AJ621250.1
	R: TCTTGCAGTCGGTCATGTTGTC		
β-Actin	F: CAACACAGTGCTGTCTGGTGGTA	58	X00182
	R: ATCGTACTCCTGCTTGCTGATCC		

a *The listed oligonucleotides were used to analyze gene expression via real-time quantitative PCR*.

b *IFN, Interferon; IL, Interleukin*.

c *F, forward; R, reverse*.

### Statistical Analysis

The expression levels of all genes were calculated relative to the housekeeping gene (β-actin) using the LightCycler® 480 software (Roche Diagnostics) and data were analyzed by using GLM procedure of SAS (SAS Institute Inc., Cary, NC). Differences among treatment means were determined using Tukey's multiple comparison test after log transformation when error deviations did not have homogenous variance across the treatments. *P*-value of <0.05 was considered statistically significant.

## Results

### Hatchability

Hatchability was recoded on the day of the hatch. The results showed that in ovo inoculation of either PBS or lactobacilli did not influence hatchability of the chickens and 99.38% of eggs were hatched following *in ovo* injection.

### Cytokine Gene Expression in Cecal Tonsils, Spleen, and Bursa of Fabricius

**Figure 1:**
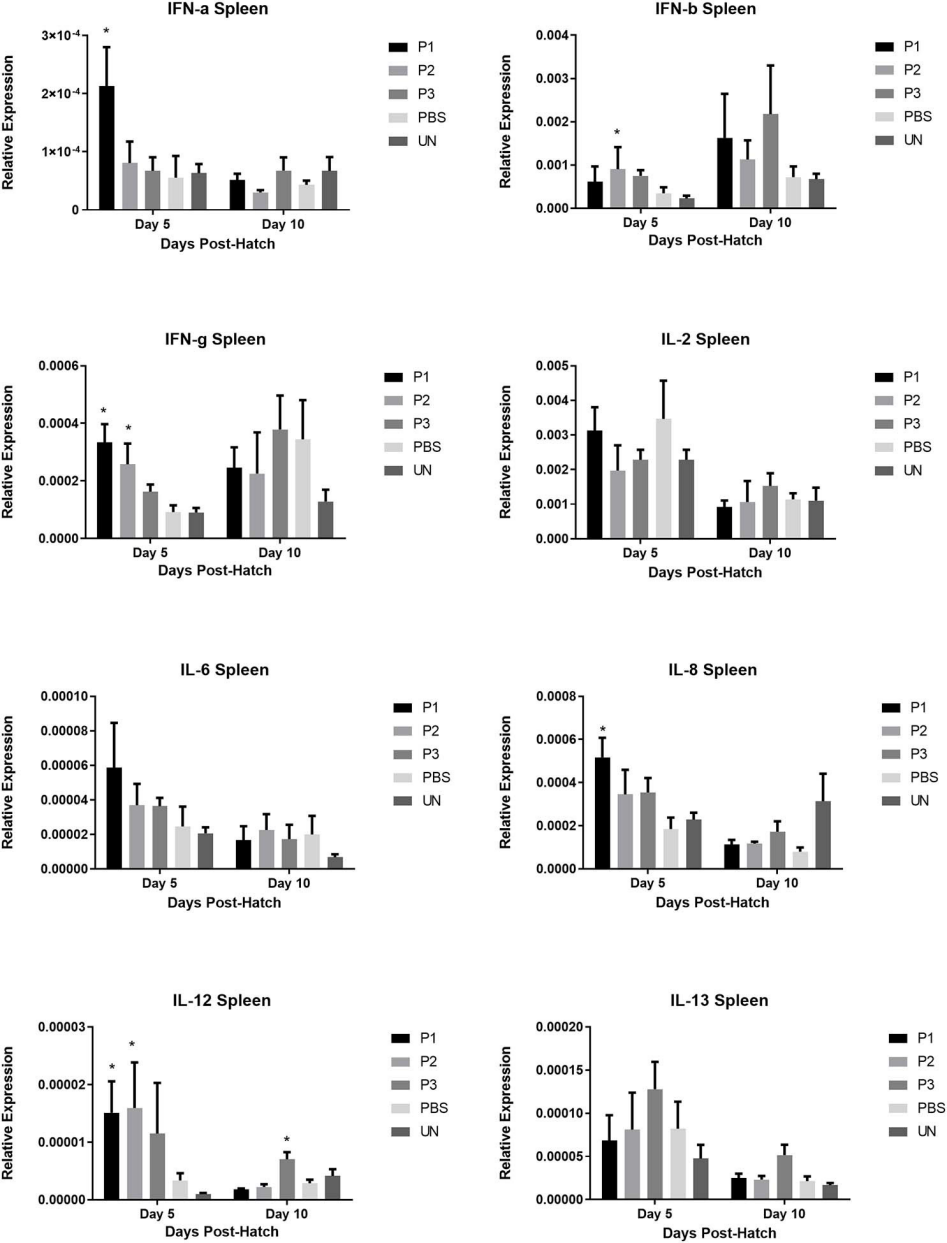
Relative gene expression of IFN-α, IFN-β, IFN-γ, IL-2, IL-6, IL-8, IL-12, and IL-13 in the spleen of chickens at days 5 and 10 post-hatch. Samples collected from 6 birds per treatment. Treatment groups were as follows: P1, P2, and P3 received 1 × 10^5^, 1 × 10^6^, 1 × 10^7^ CFU/egg of a selected mixture of *Lactobacillus* bacteria (*Lactobacillus salivarius, Lactobacillus reuteri, Lactobacillus crispatus*, and *Lactobacillus johnsonii)*, respectively (PBS, phosphate-buffered saline group; and UN, non-injected eggs). The reference gene (Beta-actin) was used for relative gene expression. Statistical significance among treatment groups was calculated using one-way ANOVA followed by Tukey's comparison test. Error bars represent standard errors of the mean. Results were considered statistically significant from the control group if *P* < 0.05. *Bars with asterisks differ significantly from control (PBS) group.

**Figure 2:**
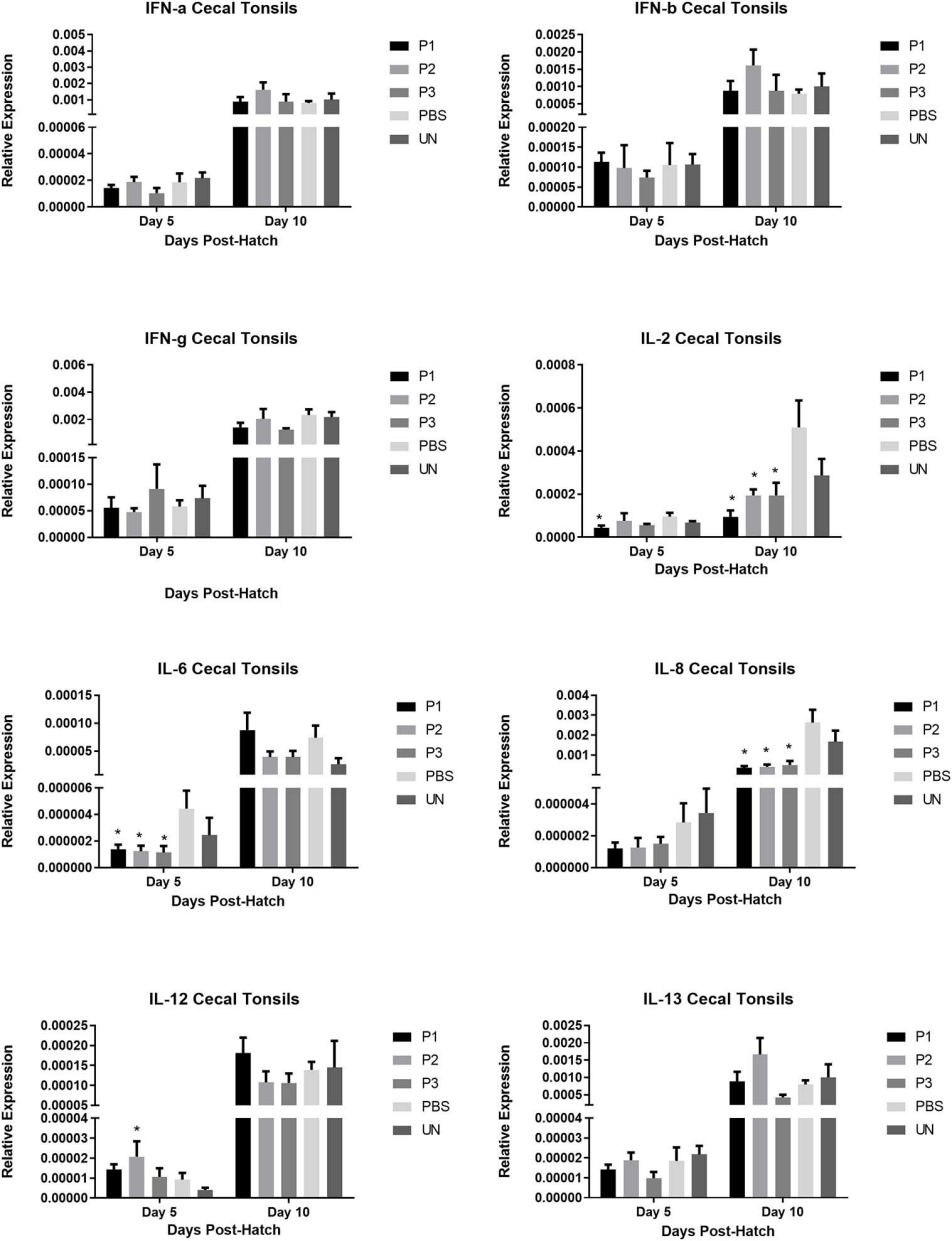
Relative gene expression of IFN-α, IFN-β, IFN-γ, IL-2, IL-6, IL-8, IL-12, and IL-13 in the bursa of Fabricius of chickens on days 5 and 10 post-hatch. Samples collected from 6 birds per treatment. Treatment groups were as follows: P1, P2, and P3 received 1 × 10^5^, 1 × 10^6^, 1 × 10^7^ CFU/egg of a selected mixture of *Lactobacillus* bacteria (*L*. *salivarius, L*. *reuteri, L*. *crispatus*, and *L. johnsonii)* respectively (PBS, phosphate-buffered saline group; and UN, non-injected eggs). The reference gene (Beta-actin) was used for relative gene expression. Statistical significance among treatment groups was calculated using one-way ANOVA followed by Tukey's comparison test. Error bars represent standard errors of the mean. Results were considered statistically significant from the control group if *P* < 0.05. *Bars with asterisks differ significantly from control (PBS) group.

**Figure 3:**
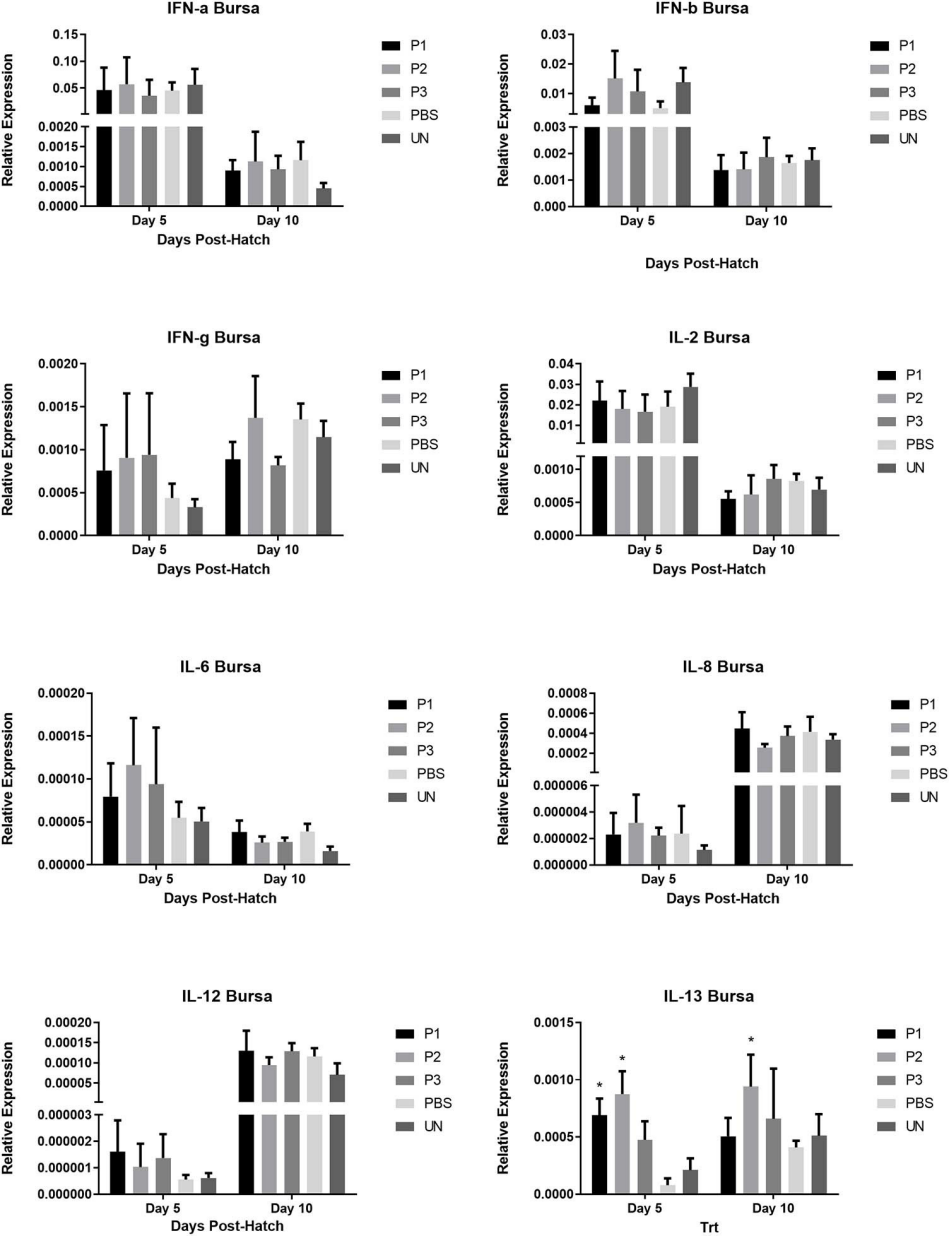
Relative gene expression of IFN-α, IFN-β, IFN-γ, IL-2, IL-6, IL-8, IL-12, and IL-13 in the cecal tonsils of chickens on days 5 and 10 post-hatch. Samples collected from 6 birds per treatment. Treatment groups were as follows: P1, P2, and P3 received 1 × 10^5^, 1 × 10^6^, 1 × 10^7^ CFU/egg of a selected mixture of *Lactobacillus* bacteria (*L*. *salivarius, L*. *reuteri, L*. *crispatus*, and *L. johnsonii)* respectively (PBS, phosphate-buffered saline group; and UN, non-injected eggs). The reference gene (Beta-actin) was used for relative gene expression. Statistical significance among treatment groups was calculated using one-way ANOVA followed by Tukey's comparison test. Error bars represent standard errors of the mean. Results were considered statistically significant from the control group if *P* < 0.05. *Bars with asterisks differ significantly from control (PBS) group.

The results for gene expression of cytokines are presented in Figures 1–3. In the spleen (Figure 1), the expression of IL-2, IL-6, and IL-13 was not altered by treatment (*P* > 0.05). However, expression of IFN***-***α, IFN*-*γ, and IL-12 on day 5 and IL-8 on day 10 post-hatch was upregulated in the spleen of birds that received 10^5^ CFU of lactobacilli (P1) *(P*< *0.05)*. In addition, lactobacilli-treatment of 10^6^ CFU (P2) significantly upregulated the expression of IFN*-*γ and IL-12 on day 5 and IFN*-*β on day 10 post-hatch. In the cecal tonsils (Figure 2), expression of IFN***-***α, IFN*-*β, IFN*-*γ, and IL-13 was not affected by lactobacilli administration (*P* > 0.05) however, it led to downregulation of IL-6 on day 5 and IL-2 and IL-8 on day 10 post-hatch. In contrast, expression of IL-12 was upregulated in lactobacilli-treated groups on days 5 (P1 and P2) and day 10 (P3) post-hatch in the cecal tonsils. No significant differences were observed in cytokine gene expression in the bursa of Fabricius, except for IL-13, which was upregulated on day 5 (P1 and P2) and on day 10 (P2) post-hatch (Figure 3).

### T Lymphocyte and Monocyte/Macrophage Populations

**Figure 4:**
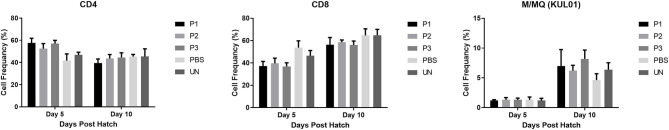
T cell subsets and monocyte/macrophage (%) in the spleen of chickens following *in ovo* inculcation of *Lactobacillus* bacteria at days 5 and 10 post-hatch. Samples collected from 6 birds per treatment. Treatment groups were as follows: P1, P2, and P3 received 1 × 10^5^, 1 × 10^6^, 1 × 10^7^ CFU/egg of a selected mixture of *Lactobacillus* bacteria (*L*. *salivarius, L*. *reuteri, L*. *crispatus*, and *L. johnsonii)* respectively (PBS, phosphate-buffered saline group; and UN, non-injected eggs). Statistical significance among treatment groups was calculated using one-way ANOVA followed by Tukey's comparison test. Error bars represent standard errors of the mean. Results were considered statistically significant from the control group if *P* < 0.05.

Results for the flow cytometric analysis KUL01 and T lymphocyte subpopulations in the spleen (CD4^+^ and CD8^+^) are presented in Figure 4. Inoculation of eggs with lactobacilli did not change the population of monocyte/macrophage and T cell subsets (single positive CD3^+^CD4^+^ and CD3^+^CD8^+^) in the spleen (*P* > 0.05).

### Anti-SRBC and Anti-KLH Antibody Titres

**Figure 5:**
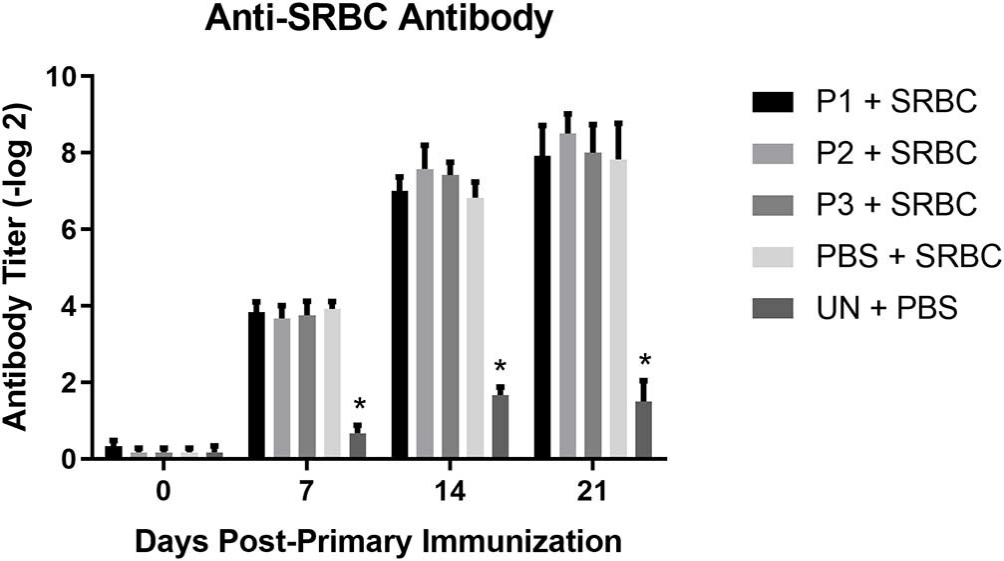
Serum anti-SRBC antibody titers as determined by direct hemagglutination assay. Treatment groups were as follows: P1, P2, and P3 received 1 × 10^5^, 1 × 10^6^, 1 × 10^7^ CFU/egg of a selected mixture of *Lactobacillus* bacteria (*L*. *salivarius, L*. *reuteri, L*. *crispatus*, and *L. johnsonii)* respectively and immunized with SRBC (P1 + SRBC, P2 + SRBC, and P3 + SRBC); chickens received 100 μl of phosphate-buffered saline/egg and were immunized with SRBC (PBS + SRBC); and chickens from non-injected eggs that were injected with PBS served as a control group (PBS). Serum samples collected from 12 birds per treatment. Statistical significance among treatment groups was calculated using one-way ANOVA followed by Tukey's comparison test. Error bars represent standard errors of the mean. Results were considered statistically significant from the control group if *P* < 0.05. *Bars with asterisks differ significantly from control (PBS) group.

The results for antibody-mediated immune responses against SRBC are presented in Figure 5. At 7, 14, and 21 days post-primary immunization, higher antibody titers against SRBC were observed in all immunized group compared to the non-immunized control group (*P* < 0.05). Nevertheless, inoculation of eggs with *Lactobacillus* bacteria did not affect serum anti-SRBC antibody titers (*P* > 0.05).

**Figure 6:**
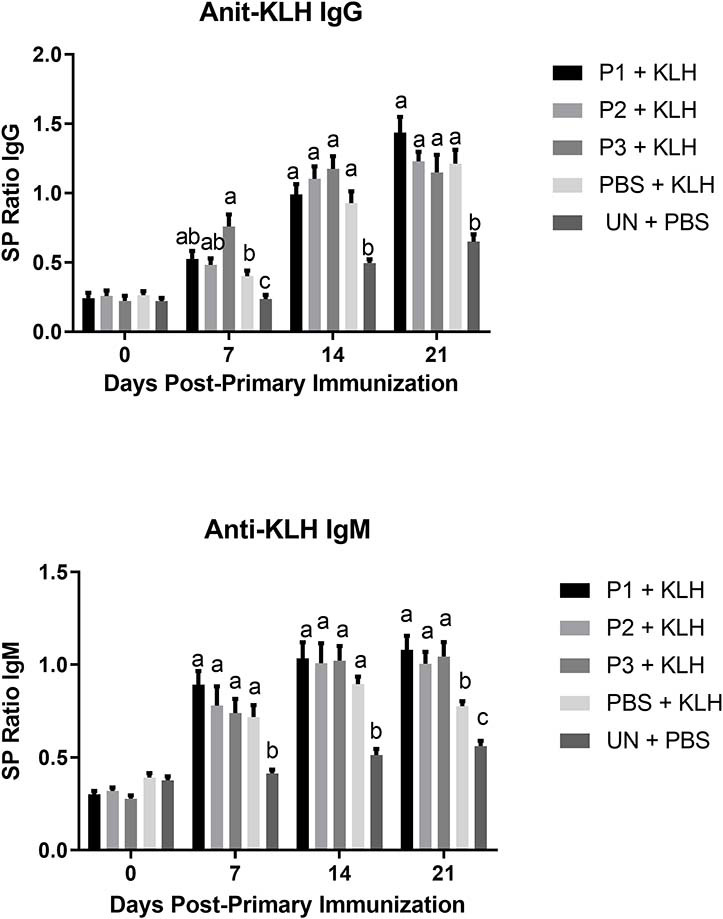
Serum anti-KLH IgG and IgM titers as determined by indirect ELISA. Treatment groups were as follow: P1, P2, and P3 received 1 × 10^5^, 1 × 10^6^, 1 × 10^7^ CFU/egg of a selected mixture of *Lactobacillus* bacteria (*L*. *salivarius, L*. *reuteri, L*. *crispatus*, and *L. johnsonii)*, respectively and immunized with KLH (P1 + KLH, P2 + KLH, and P3 + KLH); chickens received 100 μl of phosphate-buffered saline/egg and were immunized with KLH (PBS + KLH); and chickens from non-injected eggs that were injected with PBS served as control group (PBS). Serum samples collected from 12 birds per treatment. Statistical significance among treatment groups was calculated using one-way ANOVA followed by Tukey's comparison test. Error bars represent standard errors of the mean. Results were considered statistically significant from the control group if *P* < 0.05. (a–c) Means with no common superscripts differ significantly.

The results for antibody-mediated immune responses against KLH are presented in Figure 6. At 7, 14, and 21 days post-primary immunization, higher antibody titers against KLH were observed in all immunized groups compared with the non-immunized control group (*P* < 0.05). In addition, lactobacilli treatment at a dose of 10^7^ CFU (P3) significantly enhanced serum IgG and IgM titers against KLH on day 7 and day 21 post-primary immunization, respectively.

## Discussion

*In ovo* technology was first introduced to the poultry industry several decades ago for vaccination against Marek's disease virus (27). This technique enables the delivery of various pharmaceuticals and biological supplements to chicken embryos during embryonation (28). One candidate supplement that can be administered *in ovo* to provide health benefits to the chickens are probiotics. It has been reported that the gut microbiota plays a critical role in development and regulation of the immune system (29). Probiotics may enhance immune responses and control pathogen infections in chickens by improving and restoring gut microflora (30). Several studies have reported the immunomodulatory activities of probiotics in chickens (16, 26, 31, 32). Therefore, the present study was conducted to evaluate the effects of *in ovo* inoculation of lactobacilli on innate and adaptive immune responses of chickens.

In the current study, expression of IL-2 was down-regulated in the cecal tonsils of lactobacilli-treated birds. IL-2 is mainly produced by activated T lymphocytes and is involved in the proliferation and activation of both T helper and cytotoxic T cells (33). Downregulation of IL-2 in lactobacilli-treated birds suggests immunomodulatory properties of these bacteria in the absence of an infection. This suggestion can be supported by our observation that there was also a downregulation of IL-6 and IL-8 in the cecal tonsils of lactobacilli-treated birds, thus indicating that *Lactobacillus* bacteria might help maintaining immune homeostasis in the chicken intestine.

The results of previous studies indicate that dysbiosis of gut microbiota caused by a microbial challenge or an infectious disease is often associated with an activation of the immune system and upregulation of cytokines in secondary lymphoid organs in chickens (34, 35). Probiotics are thought to play a key role in maintaining the normal intestinal microbiota by reducing the population of pathogenic microorganisms though different processes, including competitive exclusion, inhibition of pathogen adhesion, and production of anti-pathogenic substances (19). In the present study, lactobacilli treatment downregulated the expression of cytokines (especially inflammatory cytokines) in cecal tonsils which are considered an intestinal lymphoid organs. This indicates that *Lactobacillus* bacteria might maintain microbial balance in the intestinal ecosystem by decreasing the population of pathogenic bacteria, thus preventing activation of the immune system. Decreased inflammatory responses to commensal bacteria within gut-associated lymphoid tissues (GALT) has been reported in previous studies, suggesting that although immune system cells in GALT can mount an inflammatory response toward pathogenic bacteria, they also remain slightly responsive to commensal bacteria (36).

Unlike in the cecal tonsils, the expression of cytokines was upregulated in the spleen, suggesting that lactobacilli might differentially modulate cytokine expression profiles in systemic (spleen) and local (cecal tonsils) secondary lymphoid organs. Gene expression in the bursa of Fabricius demonstrated that among all cytokines, only the expression of IL-13 was upregulated in lactobacilli-treated groups. Bursa of fabricius is considered as the primary lymphoid organs for B cell development and differentiation in newly hatched chick-s (37); and IL*-*13 is a T helper type 2 anti-inflammatory cytokine with the function closely related to IL-4 including stimulation of activated B cells, and differentiation of B cells into plasma cells (38). Therefore, higher expression of IL-13 in the bursa of Fabrocius of lactobacilli-treated birds suggests the role of lactobacilli as beneficial commensal bacteria in B cell development. It has been previously reported that germ-free animals show impaired immune responses against different antigens suggesting the critical role of commensal bacteria in immune system development (39). In chickens, diversification of immunoglobulin mostly occurs during embryonic development, challenging the role of microbiota in pre-hatch B cells development and Ig diversification. However, it is reported that shortly after hatch, gut microbiota appears to influence the B-lymphocyte repertoire in bursa through transepithelial pinocytotic flow of intestinal contents into bursal follicles that occurred by M cell-like follicle-associated epithelium (24, 40). To this end, our observation of augmented IL-13 expression in the bursa can imply that *in ovo* administration of probiotic lactobacilli can influence bursal development of B cells.

In this study, we evaluated the effects of a mixture of *Lactobacillus* bacteria on CD4^+^ and CD8^+^ cell populations in chicken splenocytes. T helper cells (CD4^+^) are involved in various immune system processes such as activation of B cells, macrophages and cytotoxic T cells (41). In addition, they play a key role in generating adaptive immune responses through interaction with major histocompatibility complex (MHC) class II molecules on antigen presenting cells (42). Inoculation of embryonated eggs with lactobacilli did not change the percentage of CD4^+^ splenocytes on days 5 and 10 post-hatch. In contrast, Dalloul et al. (43) demonstrated that feeding lactobacilli to chickens increased the percentage of CD4^+^ intestinal intraepithelial lymphocytes. Similarly, Noujaim et al. (22) showed that administration of a mixture of *Lactobacillus* bacteria including *L. acidophilus* and *L. reuteri* increased the number of CD4^+^ cells in the small intestine of chickens. The percentage of CD8^+^ T cells in the current study was not significantly affected by lactobacilli treatment. Asgari et al. (44) also observed no significant differences in CD8^+^ cell counts in immune system organs (cecal tonsil and bursa of fabricius) of chickens treated with lactobacilli. However, Noujaim et al. (22) demonstrated that oral treatment of *L. reuteri* and *L. acidophilus* increased the number of CD8^+^ cells in the epithelium and in the intestinal lamina propria of chickens. The inconsistent results observed in these studies could be attributed to the different types and dosages, including regimens of *Lactobacillus* bacteria in addition to differences in the route of administration used in different studies. The present results demonstrated that *in ovo* inoculation of eggs with lactobacilli enhanced serum IgG and IgM responses against KLH when a dose of 10^7^ CFU was administered. In agreement with this result, previous studies have demonstrated that dietary/oral administration of probiotic bacteria enhances antibody responses against KLH, infectious bursal disease virus and avian influenza virus (16, 44, 45). Unlike KLH, lactobacilli treatment did not affect antibody production against SRBC. Similarly, Qorbanpour et al. (46) showed that dietary supplementation with multi-strain probiotics did not change antibody production against SRBC. In contrast, other studies demonstrated that dietary or oral administration of probiotic bacteria improves antibody response to SRBC (26, 47). In another study, Brisbin et al. (16) demonstrated that oral treatment of chickens with *L. salivarius* significantly increased serum antibody responses against SRBC compared to the control group; however, no such effect was observed when chickens were treated with *L. reuteri* and *L. acidophilus*. The conflicting results regarding the effects of lactobacilli on antibody-mediated immune response observed in different studies suggests that the immunomodulatory activities of *Lactobacillus* bacteria likely cannot be generalized at this point due to a number of factors such as the strain and dose of *Lactobacillus* bacteria, administration route, immunization regimen, timing of administration and experimental conditions.

In conclusion, the results of the current study demonstrated that *in ovo* inoculation of lactobacilli downregulated cytokine gene expression in the cecal tonsils, indicating the anti-inflammatory capacity of these bacteria in the intestine. However, elevated expression of cytokines observed in the spleen of *Lactobacillus*-treated birds suggested that lactobacilli may have different immunomodulatory activities in local and systemic secondary lymphoid organs. In addition, lactobacilli-treated groups, enhanced specific antibody-mediated immune responses against a highly immunogenic T cell-dependent antigen (KLH), suggesting the stimulatory effects these bacteria have on adaptive immunity. On the other hand, *Lactobacillus* bacteria did not have significant effects on T cell subsets in the spleen. Therefore, further studies are needed to investigate the effects of *in ovo* administration of lactobacilli on T and B cells population in the local and systemic immune system organs of chickens, in addition to further exploring the protecting effects of *in ovo*-inoculated lactobacilli against challenge with an infectious pathogen.

## Data Availability Statement

All datasets generated for this study are included in the article/supplementary material.

## Ethics Statement

The animal study was reviewed and approved by Animal Care Committee, University of Guelph.

## Author Contributions

MA conceived and designed the project, collected and analyzed the data, and prepared the manuscript. BS, JA, KT-A, SK, JB, and RK helped for sample collection and reviewed the manuscripts, and provided suggestion and comments. SS provided intellectual input, approved the protocol, reviewed the manuscript and provided critical thinking, suggestion and comments.

### Conflict of Interest

The authors declare that the research was conducted in the absence of any commercial or financial relationships that could be construed as a potential conflict of interest.
