# Corrigendum: Effects of *in ovo* inoculation of multi-strain lactobacilli on cytokine gene expression and antibody-mediated immune responses in chickens

**DOI:** 10.3389/fvets.2023.1252518

**Published:** 2023-10-09

**Authors:** Mohammadali Alizadeh, Bahram Shojadoost, Jake Astill, Khaled Taha-Abdelaziz, Seyed Hossein Karimi, Jegarubee Bavananthasivam, Raveendra R. Kulkarni, Shayan Sharif

**Affiliations:** ^1^Department of Pathobiology, Ontario Veterinary College, University of Guelph, Guelph, ON, Canada; ^2^Department of Pathology, Faculty of Veterinary Medicine, Beni-Suef University, Beni Suef, Egypt; ^3^Department of Pathology and Molecular Medicine, McMaster Immunology Research Centre, M. G. DeGroote Institute for Infectious Disease Research, McMaster University, Hamilton, ON, Canada; ^4^Department of Population Health and Pathobiology, College of Veterinary Medicine, North Carolina State University, Raleigh, NC, United States

**Keywords:** lactobacilli, *in ovo*, chickens, cytokines, antibody

In the published article, there was an error in Table 1 as published. The primer sequences were mixed up during copying and pasting. The corrected Table 1 and its caption appear below.

**Table 1:** Primer sequences used for real-time quantitative PCR^a^.

**Gene^b^**	**Primer sequence^c^ (5^′^-3^′^)**	**Annealing temperature**	**GeneBank accession number**
IFN-α	F: ATCCTGCTGCTCACGCTCCTTCT	64	AB021154
	R: GGTGTTGCTGGTGTCCAGGATG		
IFN*-β*	F: GCCTCCAGCTCCTTCAGAATACG	64	AY974089
	R: CTGGATCTGGTTGAGGAGGCTGT		
IFN-γ	F: ACACTGACAAGTCAAAGCCGCACA	60	X99774
	R: AGTCGTTCATCGGGAGCTTGGC		
IL-2	F: TGCAGTGTTACCTGGGAGAAGTGGT	60	NM_204153.2
	R: ACTTCCGGTGTGATTTAGACCCGT		
IL-6	F: CGTGTGCGAGAACAGCATGGAGA	60	NM_204628.1
	R: TCAGGCATTTCTCCTCGTCGAAGC		
IL-8	F: CCAAGCACACCTCTCTTCCA	64	AJ009800
	R: GCAAGGTAGGACGCTGGTAA		
IL-12p40	F: CCAAGACCTGGAGCACACCGAAG	60	AY262752.1
	R: CGATCCCTGGCCTGCACAGAGA		
IL-13	F: ACTTGTCCAAGCTGAAGCTGTC	60	AJ621250.1
	R: TCTTGCAGTCGGTCATGTTGTC		
β-Actin	F: CAACACAGTGCTGTCTGGTGGTA	58	X00182
	R: ATCGTACTCCTGCTTGCTGATCC		

a The listed oligonucleotides were used to analyze gene expression via real-time quantitative PCR.

b IFN, Interferon; IL, Interleukin.

c F, forward; R, reverse.

The authors apologize for this error and state that this does not change the scientific conclusions of the article in any way. The original article has been updated.

